# Mitochondrial Fusion Potentially Regulates a Metabolic Change in Tibetan Chicken Embryonic Brain During Hypoxia

**DOI:** 10.3389/fcell.2021.585166

**Published:** 2021-02-09

**Authors:** Qiguo Tang, Cui Ding, Qinqin Xu, Ying Bai, Qiao Xu, Kejun Wang, Meiying Fang

**Affiliations:** Department of Animal Genetics and Breeding, National Engineering Laboratory for Animal Breeding, MOA Laboratory of Animal Genetics and Breeding, College of Animal Science and Technology, China Agricultural University, Beijing, China

**Keywords:** Tibetan chicken, energy metabolism, mitochondria, hypoxia adaptation, embryonic development

## Abstract

The Tibetan chickens (*Gallus gallus*; TBCs) are an indigenous breed found in the Qinghai-Tibet Plateau that are well-adapted to a hypoxic environment. As of now, energy metabolism of the TBCs embryonic brain has been little examined. This study investigated changes in energy metabolism in TBCs during hypoxia, and compared energy metabolism in TBCs and Dwarf Laying Chickens (DLCs), a lowland chicken breed, to explore underlying mechanisms of hypoxia adaptation. We found TBCs exhibited decreased oxygen consumption rates (OCR) and ATP levels as well as an increased extracellular acidification rate (ECAR) during hypoxia. Nevertheless, OCR/ECAR ratios indicated aerobic metabolism still dominated under hypoxia. Most important, our results revealed significant differences in TBCs brain cellular metabolism compared to DLCs under hypoxia. Compared to DLCs, TBCs had higher OCR and TCA cycle activities during hypoxia. Also, TBCs had more mitochondrial content, increased mitochondrial aspect ratio and MFN1, MFN2, and OPA1 proteins which have previously been reported to control mitochondrial fusion were expressed at higher levels in TBCs compared to DLCs, suggesting that TBCs may regulate energy metabolism by increasing the level of mitochondrial fusion. In summary, TBCs can reduce aerobic metabolism and increase glycolysis to enable adaptation to hypoxia. Regulation of mitochondrial fusion via MFN1, MFN2, and OPA1 potentially enhances the ability of TBCs to survive on the Qinghai-Tibet Plateau.

## Introduction

The Tibetan chickens (*Gallus gallus*; TBCs) are widely distributed at altitudes of 2,200–4,100 m in the Qinghai-Tibet Plateau. This unique breed has been present in the region for at least 1,000 years. Through a long period of natural and artificial selection, the appearance and physiology of TBCs have changed to adapt to conditions of extremely high altitude, low oxygen, and cold. Compared with lowland chickens, TBCs are smaller, have an enhanced blood oxygen transport capacity due to an increased number of red blood cells, lower arterial oxygen partial pressure, lower venous blood pH, and higher hemoglobin concentration (Wei et al., 2007; Zhang et al., 2007; 2012).

One of the biggest challenge for TBCs living on the Qinghai-Tibet Plateau is insufficient oxygen. Oxygen availability influences embryonic growth and development, and is an especially important factor for animals. Hypoxia imposes severe constraints on aerobic metabolism. Cells in this state are unable to acquire enough energy to maintain basic life activities and suffer a variety of serious problems (Grocott et al., 2007; Storz et al., 2007). For chicken, oxygen concentration is a critical factor affecting hatching success, and if increased during incubation can dramatically improve hatchability (Davis, 1955; Visschedijk, 1985; Altimiras and Phu, 2000). In avian species, hypoxia during incubation inhibits embryonic development and even causes damage to the development of some organs, especially the brain (Azzam and Mortola, 2007; Azzam et al., 2007; Ophelders et al., 2016; Veenith et al., 2016). The avian brain is an organ that develops earlier at the embryonic stage and the development of the brain plays a key role in the development of the entire embryo (Romanoff, 1960). The oxygen consumed in chicken brain is not accurately studied, but in adults, the brain accounts for about 2% of body weight but consumes 20% of the total oxygen consumed by the body under normoxia (Schönfeld and Reiser, 2013; Raichle, 2015). As the highest nerve center and most oxygen-sensitive organ, it is important to understand changes in brain energy metabolism under hypoxic incubation conditions, especially in the study of high-altitude hypoxia adaptation. However, the energy metabolism of TBCs embryonic brain under hypoxia still remains ununderstood.

Cellular energy metabolism is intimately linked to mitochondrial functions that play a fundamental role in providing ATP for normal brain function (Barksdale et al., 2010). Mitochondrial respiration levels are often defined using the oxygen consumption rate (OCR; Brand and Nicholls, 2011), which can be determined under various conditions and treatments using a Seahorse Extracellular Flux Analyzer to measure mitochondrial bioenergetics. Mitochondria are essential organelles that continually undergo fusion and fission to govern mitochondrial function (Okamoto and Shaw, 2005; Hoppins et al., 2007; Suen et al., 2008). These opposing processes work in concert to maintain the shape, size, and number of mitochondria and their physiological function (Chan, 2012), and mitochondrial dynamics contribute to mitochondrial respiration (Chen et al., 2005; Zorzano et al., 2010). Dynamin-related guanosine triphosphatases (GTPases) regulate mitochondrial fusion and fission events (Chan, 2012). Mitochondrial fusion is regulated by three large GTPases. Mitofusin-1 (MFN1) and mitofusin-2 (MFN2), which are located on the mitochondrial outer membrane, are involved in early steps in membrane fusion (Koshiba et al., 2004; Song et al., 2009). Optic atrophy 1(OPA1) is associated with the inner membrane and is essential for inner membrane fusion (Meeusen et al., 2006; Song et al., 2009). Mitochondrial fission is regulated by dynamin-related protein1 (DRP1). Cells lacking MFN1 and MFN2 exhibit reduced mitochondrial respiration and mitochondrial membrane potential (Chen et al., 2005, 2010). OPA1-deficient cells exhibit reduced oxidative phosphorylation (OXPHOS; Griparic et al., 2004; Zanna et al., 2008), while oxidative phosphorylation is enhanced in cells that overexpress OPA1 (Civiletto et al., 2015). Mouse hepatocytes with a dominant-negative DRP1 mutation exhibit increased OCR and IMM proton leaking (Galloway et al., 2012). These results suggest that energy production and supply are closely associated with the continuous changes in mitochondrial shape that are mediated by fission and fusion.

Although TBCs have adapted well to the high altitude for a long time, the genetic basis of adaptations to hypoxia is still unclear. Study has found changing available oxygen during incubation can change chicken embryo metabolism (Richards et al., 1991). However, changes in energy metabolism of TBCs embryos brain under hypoxia remain vague. We hypothesize TBCs brain has changes in energy metabolism for hypoxia adaption during embryo development. Mitochondrial fusion may contribute to these changes, which makes TBCs have greater advantages than lowland chickens in energy metabolism to adapt to hypoxia.

## Materials and Methods

### Sample Collection

The altitude of TBCs distribution is a range from 2,200 to 4,100 m, we simulated the 13% O_2_ of approximately 4,000 m above sea level as hypoxia incubation. Since, embryonic death peaks at two critical periods of breathing pattern changes during the incubation of chicken embryos (Hamburger and Hamilton, 1951). The first peak is during pre-incubation, covering the 4–10 day hatching period, and the other is at 15–20 days of incubation (Kuurman et al., 2001). Therefore, embryonic whole brain tissue at three stages of embryonic development (on days 8, 12, and 18 of incubation) are collected for next research. Fertilized eggs from TBCs and DLCs were collected at the Experimental Chicken Farm at China Agricultural University (CAU) and were transferred to normoxia (21% O_2_) and hypoxia (13% O_2_) incubators. One hundred eggs were incubated from each breed per condition. Temperature was maintained at 37.8°C with a relative humidity of 60%.

### Total RNA Extraction and Quantitative qRT-PCR Analysis

Six eggs were collected from each breed and oxygen condition after 8, 12, and 18 days of incubation. Total RNA was extracted from each sample using the TRIzol reagent (Invitrogen, CA, USA). cDNA was synthesized using the Fast Quant RT Kit (with gDNase) (Tiangen Biotech Co., Ltd, Beijing, China). Beta-actin was used as an endogenous control for normalization. qRT-PCR amplification was performed using primer pairs specific for target genes (Table 1). The cycling parameters used for qPCR amplification were as follows: initial heat denaturation at 95°C for 15 min, 40 cycles at 95°C for 30 s, 60°C for 30 s, and 72°C for 30 s; and a final extension at 72°C for 5 min. A melting curve analysis was performed to exclude genomic DNA contamination and to confirm primer specificities. Relative mRNA levels were calculated using the 2^−ΔΔCT^ method.

**Table 1:** Primer sequences used to amplify target genes using by quantitative real-time polymerase chain reaction (qRT-PCR).

**Name**	**Size (bp)**	**Primer sequence**	**Tm (^**°**^C)**
MFN1	294	F:5′-TGAGCATGTAGCAACGGAAG-3'	59
		R: 5′-AGCAAGCTGATTGACGGTCT-3'	
MFN2	119	F:5′-TACCAGGCAGATTTCCATCC-3'	58
		R: 5′-GTGATTGCATTGGAACAACG-3'	
OPA1	163	F:5′-TTCCTGATGCCGATGACCT-3'	57
		R: 5′-GCTGTTTCTCCTGGAGTACCTAA-3'	
DRP1	120	F:5′-GGCAGTCACAGCAGCTAACA-3'	58
		R: 5′-GCATCCATGAGATCCAGCTT-3'	
HK-1	118	F:5′-ACCTGTGCTTATGAAGACCCAAACTG-3'	59
		R: 5′-TCCGTCCTTGCTCACCATCCA-3'	
β-actin	196	F:5′-TTGTGCGTGACATCAAGGAGAAGC-3'	58
		R: 5′-CCACAGGACTCCATACCCAAGAAAGAT-3'	

*β-actin for the housekeeping as an internal control*.

### Protein Extraction and Western Blots

Brains from chicken embryos obtained on days 8, 12, and 18 of incubation were dissected in cold PBS (Gibco). Brains were homogenized in RIPA lysis buffer on ice for 30 min with a protease inhibitor cocktail (Thermo Fisher™), and centrifuged at 12,000 g for 0.5 h. Samples were boiled and denatured in loading buffer containing 5% 2-mercaptoethanol (Invitrogen) and subjected to electrophoresis through a 10% SDS polyacrylamide gel. The separated proteins were transferred to a PVDF membrane (Bio-Rad) and probed with primary antibodies for MFN1 (1/600, Proteintech), MFN2(1/600, Proteintech), OPA1 (1/600, Proteintech), DRP1 (1/800, Proteintech), α-Tubulin (1/6,000, Abcam) and the appropriate secondary antibody conjugated with horseradish peroxidase (1/5,000, Abcam). Bound proteins were detected using a Pierce ECL Western Blotting Substrate kit (Thermo Scientific™). The results were visualized by scanning and then analyzed using Image J software (NIH).

### Isolation and Culture of Embryonic Primary Brain Cells

Primary brain cells were isolated from chicken embryos on days 8, 12, and 18 of incubation. Intact brains were removed under sterile conditions and cleaned in pre-cooled Hanks solution (1× Hanks' balanced salt solution [HBSS] containing 0.5 mmol/L EDTA, and 5 mmol/L HEPES, pH 7.2, Nalgene). Brains were cut into pieces and digested with 0.125% trypsin (Gibco) for 30 min at 37°C. The digestion was terminated by addition of Fetal Bovine Serum (FBS). The mixture was fully suspended with inoculation fluid (containing 87% neurobasal™ media, 10% FBS, 2% B27, and 1% Penicillin-Streptomycin) and filtered through a 300-mesh sterile stainless steel sieve (BD Biosciences) into a 15 ml centrifuge tube (Corning) and then centrifuged for 8 min at 800 rpm. After removing the supernatant, cells were re-suspended and plated onto Poly-lysine–coated (100 μg/ml) Seahorse XF24 cell culture microplates (Seahorse Bioscience) at 10^6^ cells/plate. After cultured 3 h, the inoculation fluid was replaced with serum-free medium (containing 96% neurobasal™ media, 1% glutamine, 2% B27, and 1% Penicillin-Streptomycin). FBS, neurobasal™ media, B27, Penicillin-Streptomycin, and glutamine were all purchased from Gibco (Life Technologies, Waltham, MA, USA).

### Immunofluorescence and EdU Assay

Primary brain cells were cultured for 48 h under normoxia (21% O_2_, 5% CO_2_) and hypoxia (1% O_2_, 5% CO_2_) for immunofluorescence assay. Cells were fixed for 10 min in 4% paraformaldehyde at room temperature then 0.1% TritonX-100 for 10 min. After blocked for 1 h using 1% BSA, cells were labeled with primary antibodies (HIF1α and MAP2, 1/250) for overnight at 4°C. Next day, cells were washed 3 times with 1xPBS and then stained with respective Alexa flour 488 secondary antibody (1/250) for 1 h at room temperature. Cells were then washed and 10 mg/ml Hoechst was added for 15 min. For EdU assay, primary brain cells were cultured for 24 and 48 h under normoxia (21% O_2_, 5% CO_2_) and hypoxia (1% O_2_, 5% CO_2_). Cells were cultured in fresh growth medium which contained 10 mM EdU for 24 h. Then, the cells were fixed, permeabilized, and stained following the manufacturer's instruction (BeyotimeBio, China) and the EdU-stained cells were observed using the fluorescent microscope.

### OCR and ECAR Measurement Using Seahorse Cellular Flux Assays

Primary brain cells were cultured for 48 h under normoxia (21% O_2_, 5% CO_2_) and hypoxia (1% O_2_, 5% CO_2_) for metabolic flux analysis. The extracellular acidification rate (ECAR) and oxygen consumption rate (OCR) were measured using a Seahorse XF24 Extracellular Flux Analyzer (Seahorse Bioscience). The medium was supplemented with 10 mM glucose, 1 μM oligomycin, 100 mM 2-deoxyglucose (2-DG), 1 μM carbonyl cyanide-4-(trifluoromethoxy) phenylhydrazone (FCCP), 1 μM antimycin A, and 1 μM rotenone (final concentrations). OCR and ECAR values were normalized to the total protein per well (BCA assay).

### Citrate Synthase and α-KGDH Activity Assay

The activities of α-Ketoglutarate dehydrogenase (α-KGDH) and citrate synthase (CS) were measured using commercial assay kit from Nanjing Jiancheng Bioengineering Institute and Solarbio (China).

### Sample Preparation for GC-MS and Metabolomics Analysis

After grinding and homogenizing the whole brain in liquid nitrogen, each 40 mg sample was cooled in an ice bath and homogenized for 3 min at 30 Hz in 800 μL chloroform/methanol/water solvent (v/v/v = 2:5:2), using a Tissue Lyser (JX-24, Jingxin, Shanghai) with zirconia beads. Following centrifugation at 14,000 × g at 4°C for 15 min, 160 μL of supernatant was mixed with 10 μL deionized water containing glucose-^13^C_6_ (50 μg/mL) and evaporated to dryness under a nitrogen stream. The residue was reconstituted in 30 μL of 20 mg/mL methoxyamine hydrochloride in pyridine, and the resulting mixture was incubated at 37°C for 90 min. A 30 μL aliquot of BSTFA (with 1% TMCS) was added to the mixture. The reaction was conducted at 70°C for 60 min prior to GC-MS metabolomics analysis.

GC-MS analysis was performed on an Agilent 7890A gas chromatography system coupled to an Agilent 5975C inert MSD system (Agilent Technologies Inc., CA, USA). An OPTIMA® 5 MS Accent fused-silica capillary column (30 m × 0.25 mm × 0.25 μm; Macherey-Nagel, Düren, Germany) was utilized to separate the derivatives. Helium (>99.999%) was used as a carrier gas at a constant flow rate of 1 mL/min through the column. The injection volume was 1 μL and the solvent delay time was 6 min. Oven temperature was initially 70°C for 2 min, ramped to 160°C at a rate of 6°C/min, to 240°C at a rate of 10°C/min, to 300°C at a rate of 20°C/min, and finally held at 300°C for 6 min. The temperatures of injector, transfer line, and electron impact ion source were set to 260, 250, and 230°C, respectively. The impact energy was 70 eV, and data was collected in both full scan and SIM mode (m/z 50–600).

### Confocal Microscopy

Live-cell imaging used Nikon A1HD25 confocal microscope equipped with a 60x1.3NA oil immersion objective. Primary brain cells were stained with 20 nM MitoTracker Green (Thermo Fisher Scientific, Inc.) for 15 min and 10 mg/ml Hoechst for 20 min at 37°C. Fluorescence was excited with a 405 nm laser diode (Hoechst) and a 488 nm Argon laser.

### Image Analysis

Mitochondrial aspect ratio (the ratio of length/width) and content (% of mitochondrial area compared to whole-cell area) were quantified using Image J (NIH) according to previously described (Song et al., 2015).

### Statistical Analysis

Significance was analyzed using one-way analysis of variance (ANOVA) to test homogeneity of variances via Levene's test, followed by Student's *t*-test. Calculations and figures were plotted using Prism 7.0 (GraphPad Software Inc., San Diego, CA, USA). Differences were considered to be statistically significant for *p* < 0.05. Scale bars show the SEM of at least three separate experiments.

## Results

### Identification and Isolation of Primary Brain Cells

**Figure 1:**
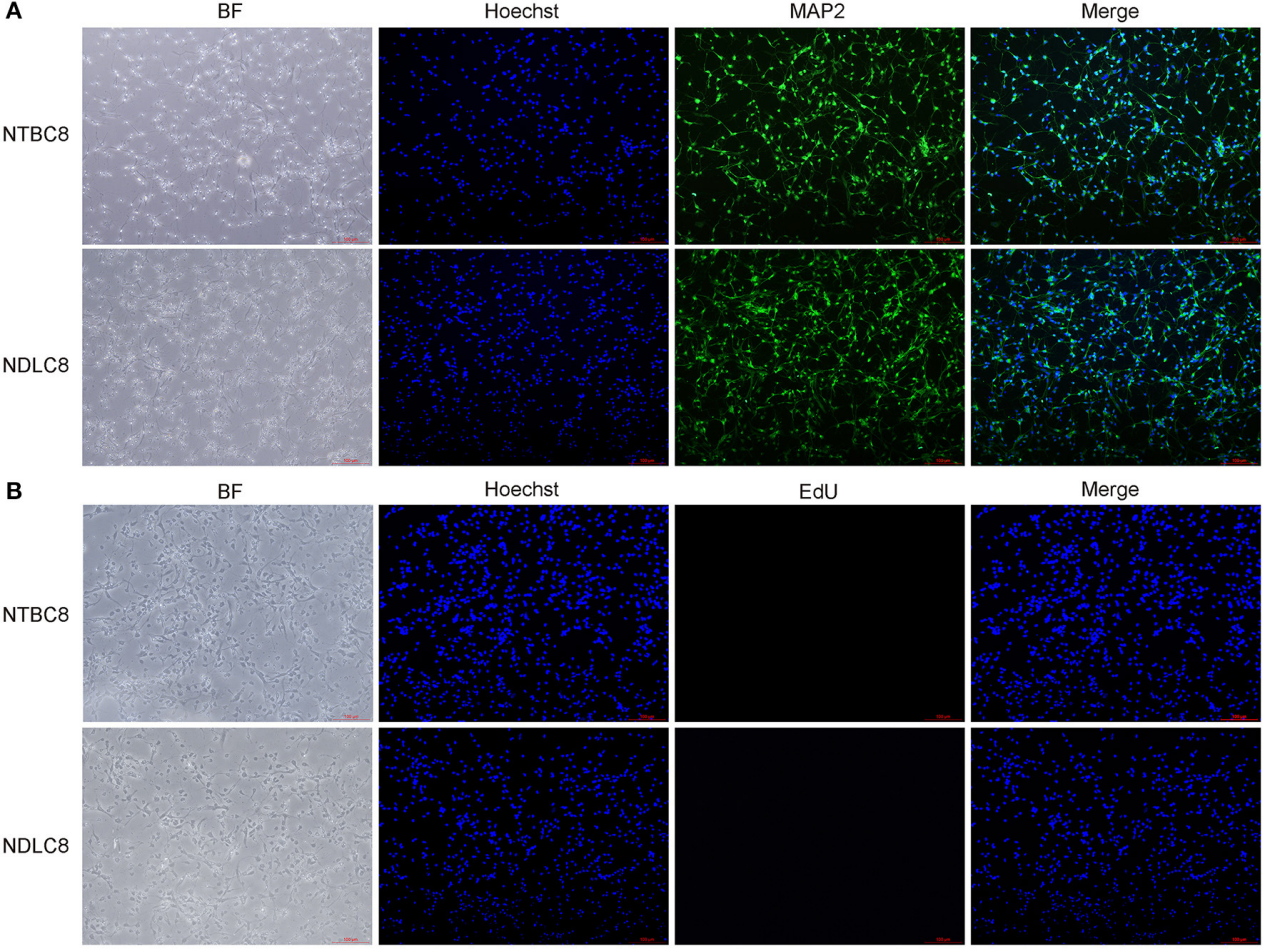
Identification **(A)** and proliferation assay **(B)** of primary brain cells. Representative images of chicken primary brain cells on day 8 of incubation cultured for 48 h.

Primary brain cells were first isolated from chicken embryos on days 8, 12, and 18 of incubation and cultured. To determine what are exactly the cells isolated from the embryonic brains, we performed the immunofluorescence assay. Cells were labeled with microtubule-associated protein 2 (MAP2), a neuron-specific cytoskeletal protein expressed in the soma and dendrites of mature neurons (Matus, 1990) and Hoechst. Result illustrated the vast majority of cells were neurons (Figure 1A). Furthermore, the cell proliferation was detected using EdU assay and most cells were not proliferating (Figure 1B).

### Aerobic Metabolism Decreases and Glycolysis Increases Under Hypoxia in TBC Embryonic Primary Brain Cells

**Figure 2:**
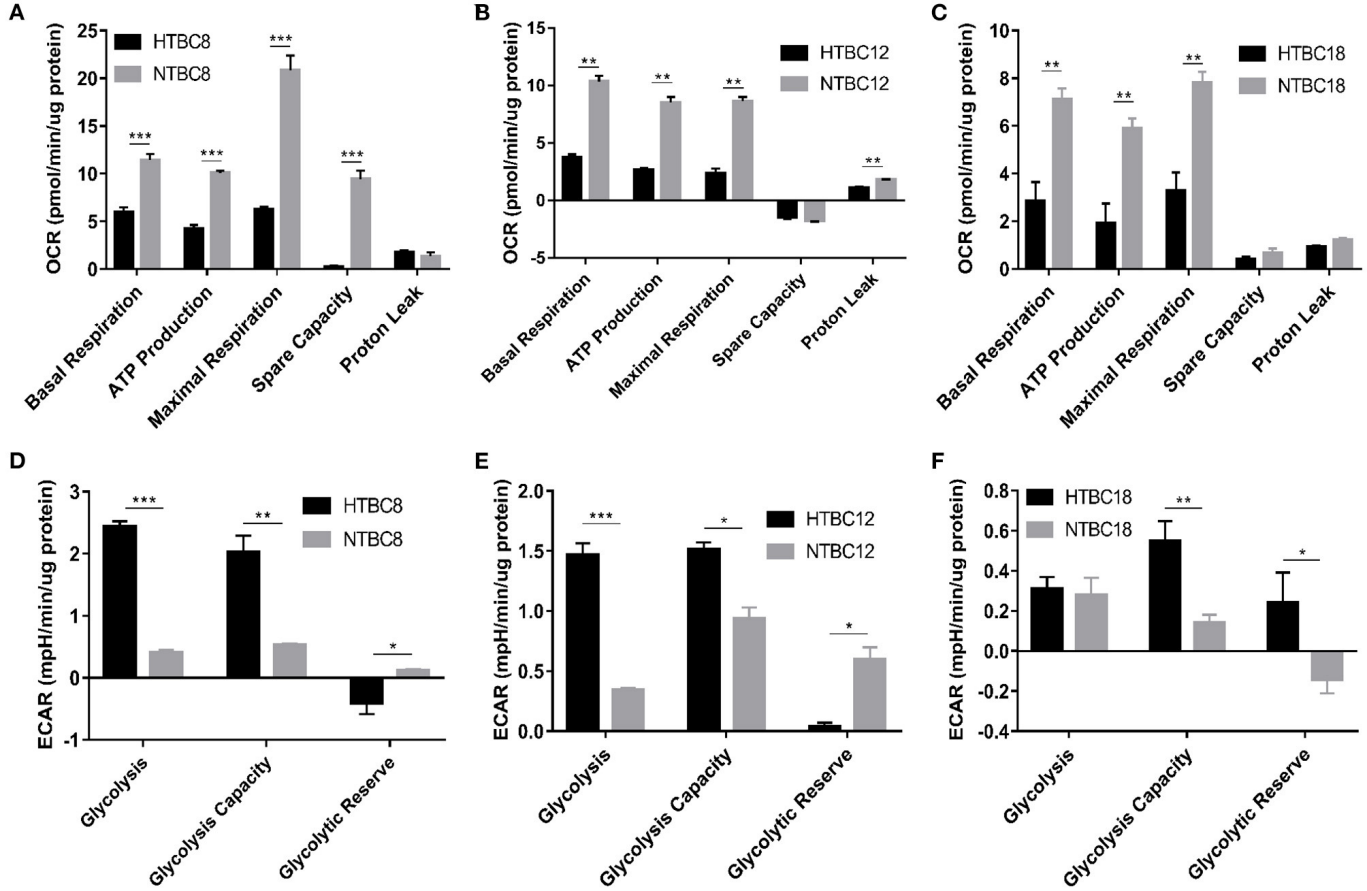
The OCR and ECAR results at different embryo developmental stages of TBCs. **(A–C)** The OCR of TBC primary brain cells on days 8,12 and 18 of incubation under hypoxia and normoxia (*n* = 3). **(D–F)** The ECAR of TBC primary brain cells on days 8, 12, and 18 of incubation under hypoxia and normoxia (*n* = 3). HTBC, TBCs under hypoxia; NTBC, TBCs under normoxia. Data are indicated as the mean ± SEM. Asterisks represent significance compared to normoxia. *P < 0.05, **P < 0.01, ***P < 0.001.

To study the energy metabolism of TBCs, we detected the energy metabolism change of TBCs embryonic primary brain cells between hypoxia and normoxia. To determine the basal bioenergetic state of the TBCs brain, a Seahorse XF24 Analyzer was used to measure OCR and ECAR. OCR measurements were obtained following sequential addition of the mitochondrial respiration inhibitors oligomycin, FCCP, and a combination of antimycin A and rotenone (Supplementary Figure 1A). A glycolysis stress test was performed by measuring ECAR in real time following the sequential addition of glucose, oligomycin, and 2-deoxyglucose (2-DG; Supplementary Figure 1B). OCR and ECAR values were normalized to the total protein per well. OCR results are presented in units of pmoles min^−1^μg^−1^ protein. ECAR results are presented in units of mpH min^−1^μg^−1^ protein. We compared OCR and ECAR in TBCs primary brain cells at different developmental stages under normoxia and hypoxia (Figure 2). At all three stages, primary brain cells exhibited significant decreases in basal respiration, ATP production, and maximal respiration in response to hypoxia. Spare respiration capacity was also significantly lower on day 8 under hypoxia (Figure 2A). Finally, proton leak was significantly lower under hypoxia on day 12 (Figure 2B), but was statistically indistinguishable on days 8 and 18 (Figures 2A,C). The ECAR results indicated that glycolysis and glycolysis capacity increased significantly under hypoxia, while glycolytic reserves decreased on days 8 and 12 (Figures 2D,E). Glycolysis capacity and glycolytic reserve differed significantly between conditions on day 18, but glycolysis did not (Figure 2F).

**Figure 3:**
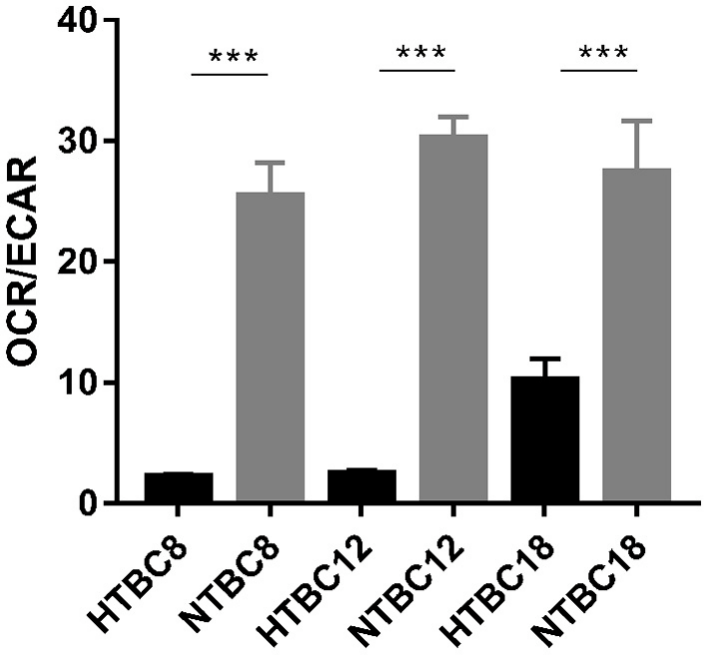
Basal bioenergetic state of TBCs at different embryo developmental stages. The basal energy metabolism was assessed by analyzing OCR/ECAR ratios on days 8, 12, and 18 of incubation (*n* = 3). HTBC, TBCs under hypoxia; NTBC, TBCs under normoxia. Data are indicated as the mean ± SEM. Asterisks represent significance compared to normoxia. ***P < 0.001.

To determine which metabolic mode dominates the TBCs brain, OCR/ECAR ratios were examined under hypoxia and normoxia. The results showed that aerobic metabolism dominated the energy supply under normoxia at different developmental stages and also under hypoxia (Figure 3). Although the OCR/ECAR ratio under hypoxia was lower than under normoxia, it was still the case that TBCs primarily rely on OXPHOS to produce ATP to meet energy needs even when oxygen was available in limited supply. Overall, these results suggest that TBCs reduce aerobic metabolism by reducing oxygen consumption rate and increasing glycolysis in response to hypoxia.

### TBCs Have Higher Energy Metabolism Than Lowland Chickens Under Hypoxia

**Figure 4:**
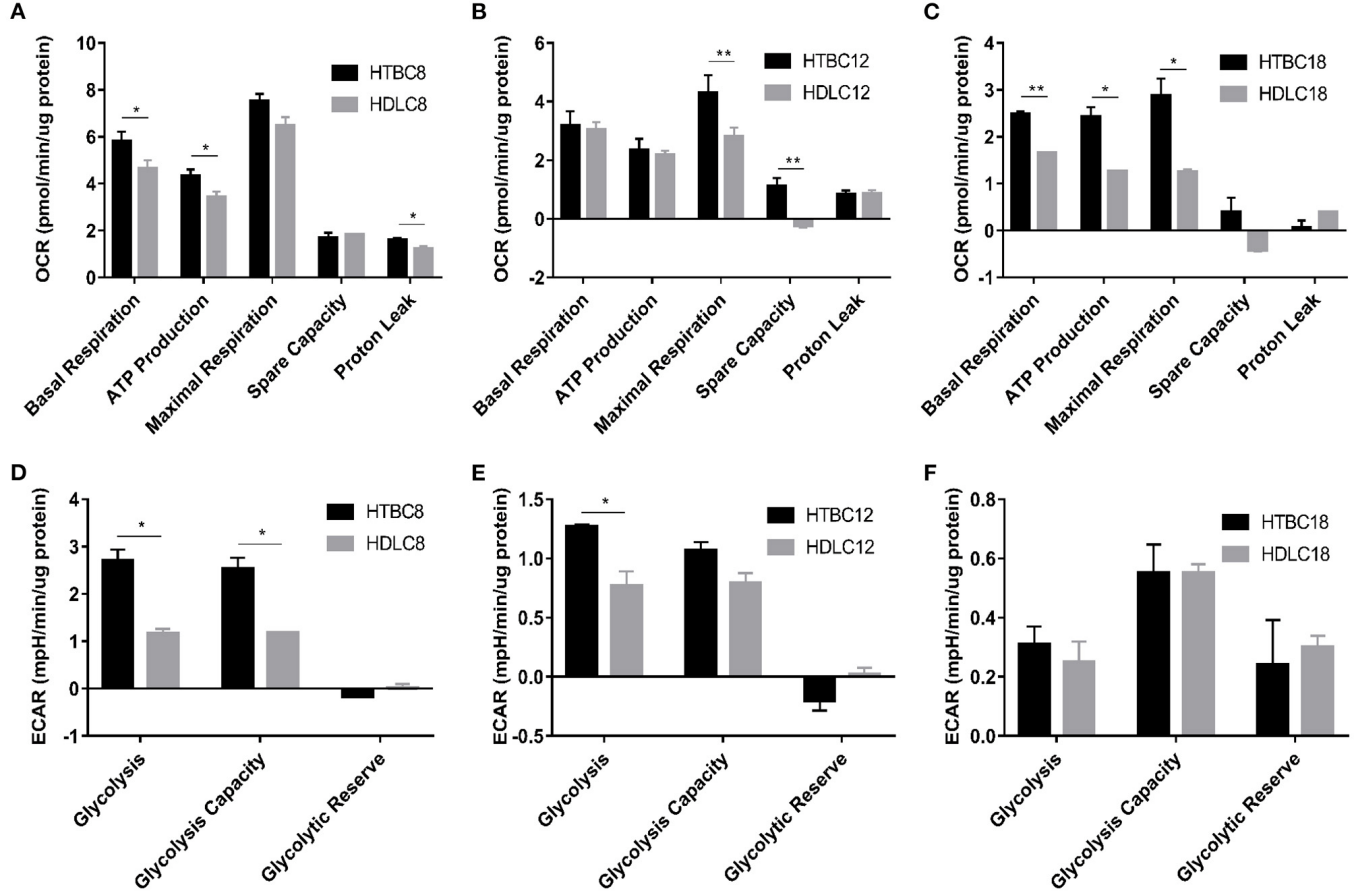
The OCR and ECAR results under hypoxia at different embryo developmental stages of chicken. **(A–C)** The OCR of chicken primary brain cells on days 8, 12, and 18 of incubation under hypoxia (*n* = 3). **(D–F)** The ECAR of chicken primary brain cells on days 8, 12, and 18 of incubation under hypoxia (*n* = 3). HTBC, TBCs under hypoxia; HDLC, DLCs under hypoxia. Data are indicated as the mean ± SEM. Asterisks represent significance TBCs compared to DLCs. *P < 0.05, **P < 0.01.

Having found TBCs reduce aerobic metabolism and increasing glycolysis responded to hypoxia, we sought to measure the difference in energy metabolism between TBCs and lowland chickens. OCR and ECAR were compared in TBCs and Dwarf Laying Chickens (DLCs). The DLCs are a dwarf laying hen found in Beijing (altitude 43.5 m). The results were shown in Figure 4 and Supplementary Figure 3. Although TBCs and DLCs differed in the Cell Mito Stress Test under normoxia, the difference was not statistically significant on days 12 and 18 (Supplementary Figures 3B,C). The exception was that ATP production was statistically significant on day 8 (Supplementary Figure 3A). Interestingly, spare capacity was higher in TBCs both on day 8 and 18 compared to DLCs. However, results from the glycolysis stress test were significantly different between TBCs and DLCs on days 8 and 18 (Supplementary Figures 3A,C). Glycolysis and glycolytic reserves in the two breeds differed significantly but opposite trends on day 8 (Supplementary Figure 3A), while glycolytic capacity and glycolytic reserve in TBCs significantly decreased on day 18 (Supplementary Figure 3C). These results show no difference in OCR between TBCs and DLCs on days 12 and 18 and significantly different in ECAR on days 8 and 18 under normoxia.

Surprisingly, we identified differences in energy metabolism between TBCs and DLCs under hypoxia (Figure 4). TBCs displayed higher basal respiration, ATP production, and proton leakage on day 8 (Figure 4A), higher maximal respiration and spare capacity on day 12 (Figure 4B), and higher basal respiration, ATP production, maximal respiration on day 18 (Figure 4C). The ECAR results in Figures 4D–F also indicated that glycolysis and glycolytic capacity increase significantly, representing increased energy production by glycolysis under hypoxia in TBCs compared to DLCs on days 8 and 12. But no difference on day 18. Together, compared to DLCs, above results suggest that TBCs obtain more ATP from aerobic metabolism, and have a superior ability to harness the rapid oxidation of substrates (such as glucose) to meet metabolic challenges in hypoxia situations.

### TBCs Have a Higher Concentration of Intermediates in the Tricarboxylic Acid Cycle (TCA Cycle) Under Hypoxia

**Figure 5:**
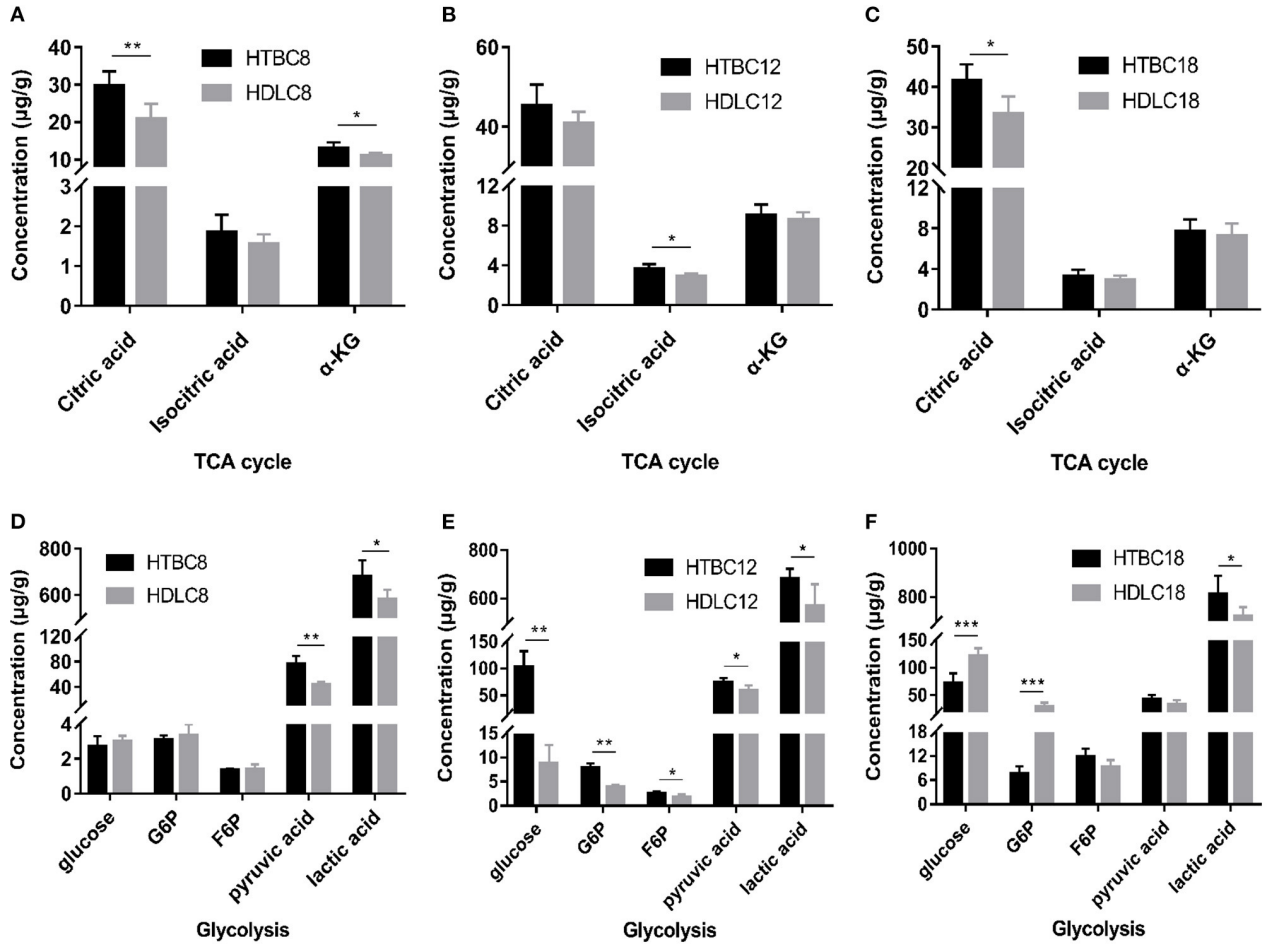
The metabolome results under hypoxia at different developmental stages of chicken. **(A–C)** The concentration of intermediate metabolites in the TCA cycle on days 8, 12, and 18 of incubation under hypoxia (*n* = 6–8). **(D–F)** The concentration of intermediate metabolites during glycolysis on days 8, 12, and 18 of incubation under hypoxia (*n* = 6–8). HTBC, TBCs under hypoxia; HDLC, TBCs under hypoxia. Data are indicated as the mean ± SEM. Asterisks represent significance TBCs compared to DLCs. *P < 0.05, **P < 0.01, ***P < 0.001.

**Figure 6:**
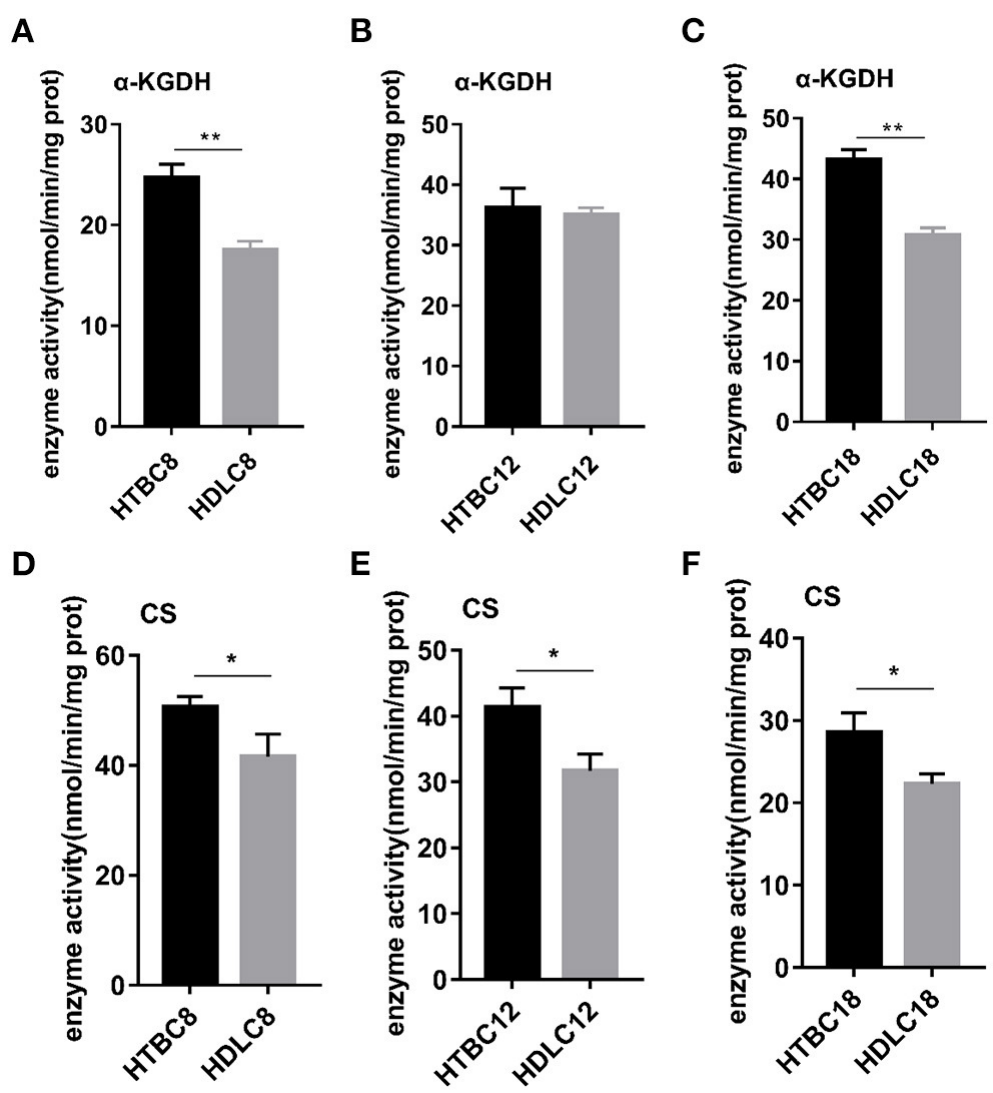
The enzyme activity results under hypoxia at different developmental stages of chicken. **(A–C)** Enzyme activity of α-KGDH on days 8, 12, and 18 of incubation under hypoxia (*n* = 3). **(D–F)** Enzyme activity of CS on days 8, 12, and 18 of incubation under hypoxia (*n* = 3). HTBC, TBCs under hypoxia; HDLC, TBCs under hypoxia. Data are indicated as the mean ± SEM. Asterisks represent significance TBCs compared to DLCs. *P < 0.05, **P < 0.01.

Because the results presented above suggest that the TBCs have a relatively higher level of aerobic metabolism than DLCs under hypoxia, we conducted experiments to determine if there are differences between TBCs and DLCs in the TCA cycle and glycolysis. A metabolome survey and enzyme activity assays were used to measure concentrations of intermediate products and activities of key enzymes (Figures 5, 6). On day 8 of incubation, TBCs and DLCs both have low levels of glucose (2.70 μg^.^g^−1^ tissue compared to 3.00 μg^.^g^−1^ tissue), but TBCs have higher levels of pyruvate (75.97 μg^.^g^−1^ tissue) and lactate (679.67 μg^.^g^−1^ tissue) (Figure 5A). Citric acid and α-ketoglutaric acid (α-KG) are also significantly different (Figure 5A). We included assay for α-ketoglutarate dehydrogenase (α-KGDH) and citrate synthase (CS), two crucial enzymes that catalyze the conversion of α-KG to succinyl-CoA and the formation of citric acid from oxaloacetate and acetyl-CoA, respectively (Figure 6). The result was consistent with those shown in Figure 4A. On day 12 of incubation, TBCs had significantly higher levels of glucose (more than 11 times) and glucose-6-phosphate (G6P, about 1.96 times), compared to DLCs (Figure 5E). Isocitrate levels in the TCA cycle of TBCs were also significantly higher, while citric acid and α-KG were statistically indistinguishable (Figure 5B). Despite this, the results still suggested that glycolysis and the TCA cycle were relatively more active in TBCs, consistent with the results shown in Figures 4B,E. On day 18 of incubation, citric acid level was significantly different in the two breeds shown in Figure 5C. Although α-KG levels were similar, α-KGDH activity differed between TBCs and DLCs (Figure 6C). Unexpectedly glucose and G6P levels were markedly higher in DLCs (Figure 5F), which seems inconsistent with the results shown in Figure 4F. However, measurements of Hexokinase 1 (*HK-1*) mRNA levels show that *HK-1* expression was significantly higher in TBCs (Supplementary Figure 4G). These results which was consistent with OCR results in Figure 4 indicate the significantly different in TCA cycle of TBCs embryonic brain compared to DLCs at different developmental stages.

### TBCs Have Higher Mitochondrial Quality Under Hypoxia

**Figure 7:**
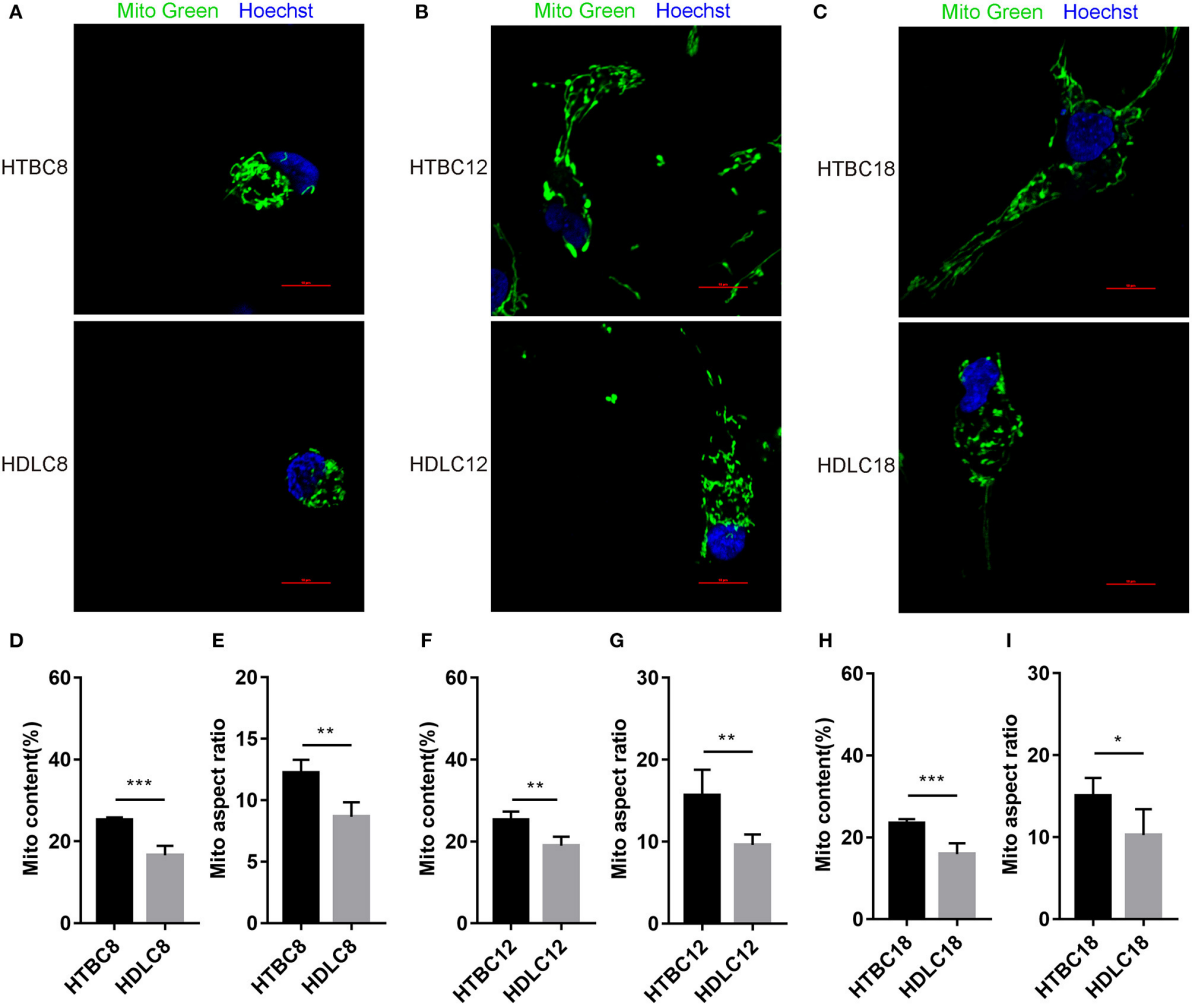
Mitochondrial quality under hypoxia at different developmental stages of chicken. **(A–C)** Mitochondria of cells stained with MitoTracker Green. **(D–I)** Quantitative mitochondrial aspect ratio and mitochondrial content in cells. *P < 0.05, **P < 0.01, ***P < 0.001.

Having observed a higher energy metabolism level in TBCs than DLCs under hypoxia, we sought to determine whether the increase is related to mitochondrial. The results demonstrated no difference in mitochondrial including mitochondrial content and mitochondrial aspect ratio between TBCs and DLCs under normoxia (Supplementary Figure 5). Then we assayed these under hypoxia, which decreased in mitochondrial content and mitochondrial aspect ratio compared to normoxia both TBCs and DLCs. But they were still higher in TBCs than DLCs (Figure 7). We also included an assay for citrate synthase (CS) which an indicator of the amount of mitochondrial mass (Figures 6D–F and Supplementary Figures 4D–F). We found no difference under normoxia between TBCs and DLCs on days 8, 12, and 18 of incubation. However, the enzyme activity was significantly higher under hypoxia in TBCs than DLCs in the three incubation stages. The above results indicate that TBCs have higher amount of mitochondrial mass under hypoxia compared to DLCs.

### Mitochondrial Fusion Potentially Regulates Mitochondrial Respiration Under Hypoxia

**Figure 8:**
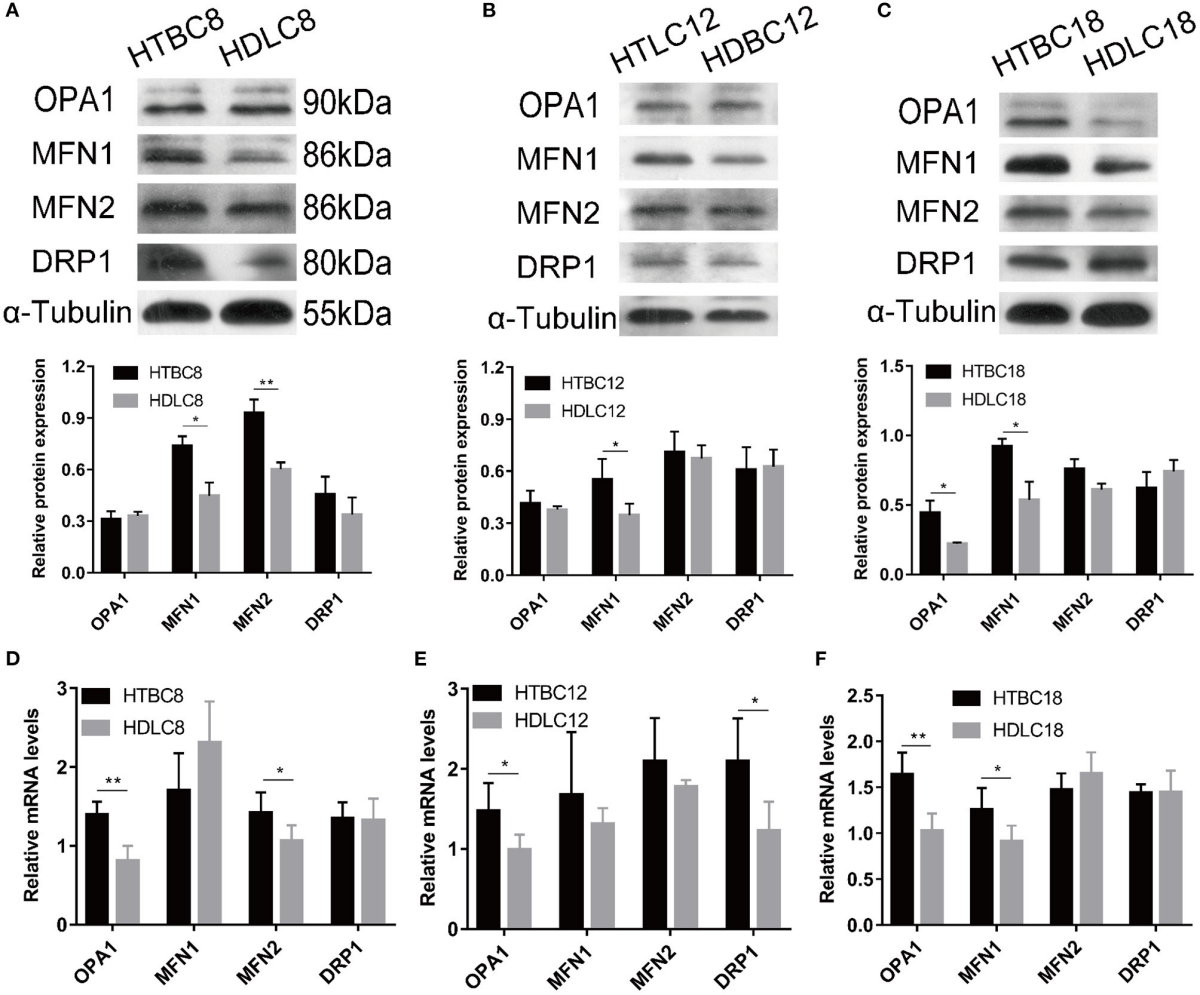
The mRNA expression and Western blot results under hypoxia at different developmental stages of chicken. **(A–C)** The Western blot on days 8, 12, and 18 of incubation under hypoxia (*n* = 3). **(D–F)** The mRNA expression on days 8, 12, and 18 of incubation under hypoxia (*n* = 6). HTBC, TBCs under hypoxia; HDLC, DLCs under hypoxia. Data are indicated as the mean ± SEM. Asterisks represent significance TBCs compared to DLCs. *P < 0.05, **P < 0.01.

Having observed a higher mitochondrial quality in TBCs than DLCs under hypoxia, we sought to determine whether it is related to mitochondrial fusion which is regulated by MFN1, MFN2, and OPA1. We performed western blotting and quantitative qRT–PCR assays to detect MFN1, MFN2, and OPA1 expression. The results suggested that mitochondrial fusion was promoted in DLCs compared to TBCs on day 8 of incubation under normoxia (Supplementary Figure 6). Surprisingly, the opposite was observed under hypoxia (Figure 7). OPA1 and MFN2 mRNA expressions were significantly more abundant while the proteins of MFN1 and MFN2 demonstrated by Western blot were significantly in TBCs compared to DLCs on day 8 (Figures 8A,D). MFN1 protein level increased, but the corresponding mRNA levels were the same in the two breeds. Similar results were observed for OPA1 and DRP1 on day 12 of incubation (Figure 8E). Since mRNA and protein expression levels for a given gene can differ widely, this discrepancy may reflect differences in regulation at the transcriptional and translational levels. We did not investigate this further, but focused primarily on protein levels because they have an immediate effect on mitochondrial regulation. The results shown in Figure 8A were consistent with those in Figure 4A, which indicated that TBCs had a higher capacity for mitochondrial respiration. Protein expressions of MFN1 were elevated in TBCs on day 12 (Figure 8B). The result suggested that mitochondrial fusion occurred more readily in TBCs and may contribute to changes in mitochondrial respiration on day 12, consistent with Figure 4B. OPA1 and MFN1 protein expressions demonstrated by Western blot (Figure 8C) were significantly more abundant in TBCs, which was consistent with the mRNA of OPA1 and MFN1 demonstrated by qRT-PCR (Figure 8F) compared to DLCs on day 18. The result was consistent with Figure 4B, which showed TBCs had higher mitochondrial respiration on day 18 compared to DLCs. The above results indicate that TBCs may have stronger mitochondrial fusion ability than DLCs to regulate energy metabolism for hypoxic adaptation.

## Discussion

Oxygen concentration gradually decreases with increasing altitude and the amount of oxygen available to animals decreases. The indigenous populations, human and animal, that inhabit the Qinghai-Tibet Plateau have adapted well to the low oxygen environment by adjusting their physiology (Wu and Kayser, 2006; He et al., 2018). Study has found TBCs have a higher embryo survival rate than lowland chickens (54.1–24.7%) under hypoxia (Wu et al., 2012). Our result also proved it that the survival rate in TBCs and DLCs was 87.14% and 89.74% under normoxia, yet 49.44 and 27.66% under hypoxia. Genomic analysis reveals that the calcium signaling pathway, which indirectly regulates energy metabolism related to the mitochondrion, is an important target for selection in hypoxic high-altitude adaptation in TBCs (Wang et al., 2015). Our study found TBCs embryonic brain reduced aerobic metabolism and increased glycolysis in response to hypoxia compared with normoxia. We measured glycolytic flux and mitochondrial respiration by using a Seahorse XF24 Analyzer, and detected intermediates of TCA cycle using GC-MS analysis with metabolomic approach. The Seahorse analysis of OCR and ECAR (Figure 2) showed a significant decrease in OCR and increase in ECAR in TBCs during hypoxia. This reflected a metabolic shift from aerobic metabolism to anaerobic metabolism. Despite the decrease in OCR/ECAR ratios due to insufficient oxygen, aerobic metabolism still dominated to supply ATP that is needed during embryonic development (Figure 3). These results demonstrate that TBCs can regulate energy metabolism to adapt to the hypoxic plateau environment. One mechanism that potentially accounts for the shift is the regulation of mitochondrial fusion (Zanna et al., 2008; Chen et al., 2010). Expression of mRNAs from the mitochondrial fusion-related genes *MFN1, MFN2*, and *OPA1* decline significantly during hypoxia (Supplementary Figure 2). This suggests that the reduction in OCR may be associated with a decrease in mitochondrial fusion.

Although decreases in OCR, ATP, and mitochondrial fusion are observed in TBCs during hypoxia compared to normoxia (Figure 3 and Supplementary Figure 2), TBCs also exhibited higher levels of aerobic metabolism than DLCs (Figure 4). Moreover, higher concentrations of the TCA cycle intermediates citrate, isocitrate, and α-KG, were observed, which suggested that TBCs compared to DLCs had a higher level of energy metabolism during hypoxia (Figure 5). In addition to generating electron carriers for the electron transport chain to yield ATP, mitochondrial citrate also regulates anabolic reactions as the carbon source for fatty acid synthesis (FAs), cholesterol, and ketone bodies (Hatzivassiliou et al., 2005; Spinelli and Haigis, 2018). α-KG is directly involved in the prolyl hydroxylase (PHD) reaction. Citrate and isocitrate can inhibit PHDs during the hypoxia response in tumor cells (Dalgard et al., 2004; Lu et al., 2005;Hewitson et al., 2007).

Compared to DLCs, TBCs embryonic brain also exhibited a higher level of glycolysis with rising OCR on days 8 and 12, which was inconsistent with the ECAR results under normoxia (Figure 5 and Supplementary Figure 3). Higher levels of pyruvate were measured during hypoxia (Figures 5D,E). Pyruvate is generated from several sources, of which glucose catabolism is the most important. Glucose catabolism is dependent on oxygen availability and mitochondrial respiratory capacity (Hui et al., 2017). Pyruvate is catabolized inside mitochondria via the TCA cycle in normoxia and is converted into lactate during hypoxia (Papandreou et al., 2006). Glucose is the primary energy substrate of the brain (Schönfeld and Reiser, 2013). Higher levels of glucose and G6P were observed in DLCs on day 18 (Figure 5F), which initially seems inconsistent with our other results. However, pyruvate and lactate levels are still higher in TBCs (Figure 5F). Glucose is phosphorylated by *hexokinase 1* (*HK1*) to produce G6P, which can be processed by glycolysis, the pentose phosphate pathway (PPP), and glycogenesis (Bélanger et al., 2011). Furthermore, *HK-1* mRNA level was significantly different in TBCs and DLCs (Supplementary Figure 4G). G6P may be involved in other metabolic processes that reduce the differences in glycolysis in DLCs and TBCs (Figure 4F), but this hypothesis has not yet been tested experimentally.

Low oxygen reduces oxidative phosphorylation and Krebs cycle rates, and both processes occur within mitochondria (Raimundo et al., 2011; Porporato et al., 2018). Mitochondria are subjected to frequent fission and fusion events, forming a highly dynamic tubular network that is modulated in response to metabolic changes in the cell (Solaini et al., 2010). Mitochondrial fusion is associated with OCR and ATP levels and is regulated by *MFN1, MFN2*, and *OPA1* (Chan, 2012). A significant increase in mitochondrial fusion occurs in DLCs under normoxia based on the protein expression of fusion-related gene *MFN2* as demonstrated by Western blot assay on day 8 (Supplementary Figure 6). Surprisingly, the opposite occurs during hypoxia. We found TBCs had higher mitochondrial quality with more mitochondrial content and higher mitochondrial aspect ratio under hypoxia. A significant increase in mitochondrial fusion occurred in TBCs compared to DLCs (Figures 7, 8), which correlated with increases in OCR and ATP levels (Figure 4) and the TCA cycle activity (Figure 5). These results are consistent with earlier reports that deletion of any of the dynamics machinery perturbs oxidative phosphorylation rate under baseline conditions (Liesa and Shirihai, 2013). MFN2 protects against neuro-degeneration in the cerebellum (Chen et al., 2007) and OPA1 is highly expressed in the brain (Delettre et al., 2001). Deletion of *MFN* genes or *OPA1* decreases cellular respiration in mouse fibroblasts and myoblasts (Bach et al., 2003; Chen et al., 2005). Furthermore, Western blot and qRT-PCR data suggested that the regulation of mitochondrial fusion was slightly different during TBCs development (Figure 8). Our study found significant differences in MFN1 and MFN2 on day 8, MFN1 on day 12, MFN1 and OPA1 on day 18 in TBCs compared to DLCs. The discrepancy may be due to fact that breathing shifts from the chorioallantoic membrane to the lung during this period, but confirmation will require additional experiments. Overall, our results suggest that mitochondrial fusion may change energy metabolism in TBCs as an adaptive strategy for hypoxia.

Besides, we also investigated HIF-1a, which was found to modulate the expression of many metabolic enzymes as well as the expression/activity of mitochondrial fusion/fission-controlling factors. More HIF-1a transferred to nuclear in TBCs under hypoxia (Supplementary Figure 7), which indicated that it may be involved in regulating the hypoxia adaptation of TBCs. The HIF-1a-mediated signaling pathway potentially involved in the hypoxic adaptation of TBCs needed further research.

We conclude that TBCs embryonic brain can reduce aerobic metabolism and increase glycolysis to enable adaptation to hypoxia. Compared to DLCs, TBCs embryonic brain have a higher energy metabolism level, which may be associated with regulation of mitochondrial fusion via MFN1, MFN2, and OPA1, to enhance the ability of TBCs to survive on the Qinghai-Tibet Plateau. We have a hypothesis that this is an important mechanism via regulating mitochondrial fusion to change metabolic status for hypoxia adaptation in TBCs.

## Data Availability Statement

The original contributions generated for the study are included in the article/Supplementary Material. The raw data supporting the conclusions of this article will be made available by the authors.

## Ethics Statement

The animal study was reviewed and approved by Experimental Animal Ethics Committee of China Agricultural University.

## Author Contributions

QT, CD, and MF conceived the study design. QT and CD conducted the experiments. QT wrote the paper. QiaX and KW helped prepare the materials. YB and QinX took part in performing the experiments. QT and MF refined the manuscript. All the authors discussed the results and improved the manuscript.

## Conflict of Interest

The authors declare that the research was conducted in the absence of any commercial or financial relationships that could be construed as a potential conflict of interest.
