# Supplementary material for: Mitochondrial Fusion Potentially Regulates a Metabolic Change in Tibetan Chicken Embryonic Brain During Hypoxia
Source: Front Cell Dev Biol. 2021 Feb 9;9:585166. doi: 10.3389/fcell.2021.585166 (PMC7900496; doi:10.3389/fcell.2021.585166)
Supplement: Supplementary file 1 [file Table_1.DOCX]

**Supplementary Figure Legends**

**Figure S1.** Agilent Seahorse XF Stress Test profile of the key. (A) Agilent Seahorse XF Cell Mito Stress Test profile of the key parameters of mitochondrial respiration (User Guide Kit 103015-100, Agilent). (B) Agilent Seahorse XF Glycolysis Stress Test profile of the key parameters of glycolytic function (User Guide Kit 103020-100, Agilent).

**Figure S2.** mRNA expression of mitochondrial fusion-related genes at different developmental stages of TBCs. (A-C) The mRNA expression of *MFN1, MFN2, OPA1* and *DRP1* on days 8,12 and 18 of incubation(n=6). NTBC: TBCs under normoxia. HTBC: TBCs under hypoxia. Data are indicated as the mean ± SEM. Asterisks represent significance compared to normoxia. *P < 0.05, **P < 0.01, ***P < 0.001.

**Figure S3.** The OCR and ECAR results under normoxia at different developmental stages of chicken. (A-C) The OCR and ECAR of chicken primary brain cells on days 8,12 and 18 of incubation under normoxia(n=3). NTBC: TBCs under normoxia. NDLC: DLCs under normoxia. Data are indicated as the mean ± SEM. Asterisks represent significance compared to normoxia. *P < 0.05, **P < 0.01.

**Figure S4.** Enzyme activity under hypoxia at different embryo developmental stages of chicken and mRNA expression of HK-1 on day 18. (A-C) Enzyme activity of α-KGDH of chicken primary brain cells on days 8,12 and 18 of incubation under normoxia(n=3). (D-E) Enzyme activity of CS of chicken primary brain cells on days 8,12 and 18 of incubation under normoxia(n=3). (G) Relative mRNA level of *HK-1* on day 18 under hypoxia(n=6). NTBC and HTBC: TBCs under normoxia and hypoxia. NDLC and HDLC: DLCs under normoxia and hypoxia. Data are indicated as the mean ± SEM. Asterisks represent significance TBCs compared to DLCs. *P < 0.05, **P < 0.01.

**Figure S5.** Mitochondrial quality under normoxia at different developmental stages of chicken. (A-C) Mitochondria of cells stained with MitoTracker Green. (D-I) Quantitative mitochondrial aspect ratio and mitochondrial content in cells. *P < 0.05, **P < 0.01, ***P < 0.001.

**Figure S6.** The RT-qPCR and Western blot under normoxia at different embryo developmental stages between TBCs and DLCs. (A-C) The mRNA(n=6) and protein(n=3) expression levels on days 8,12 and 18 of incubation under normoxia. NTBC: TBCs under normoxia. NDLC: DLCs under normoxia. Data are indicated as the mean ± SEM. Asterisks represent significance TBCs compared to DLCs. *P < 0.05, **P < 0.01.

**Figure S7.** Immunofluorescence staining of HIF-1a in cells under hypoxia at different developmental stages of chicken.
