# Supplementary figures and images for: Mitochondrial Fusion Potentially Regulates a Metabolic Change in Tibetan Chicken Embryonic Brain During Hypoxia
Source: Front Cell Dev Biol. 2021 Feb 9;9:585166. doi: 10.3389/fcell.2021.585166 (PMC7900496; doi:10.3389/fcell.2021.585166)

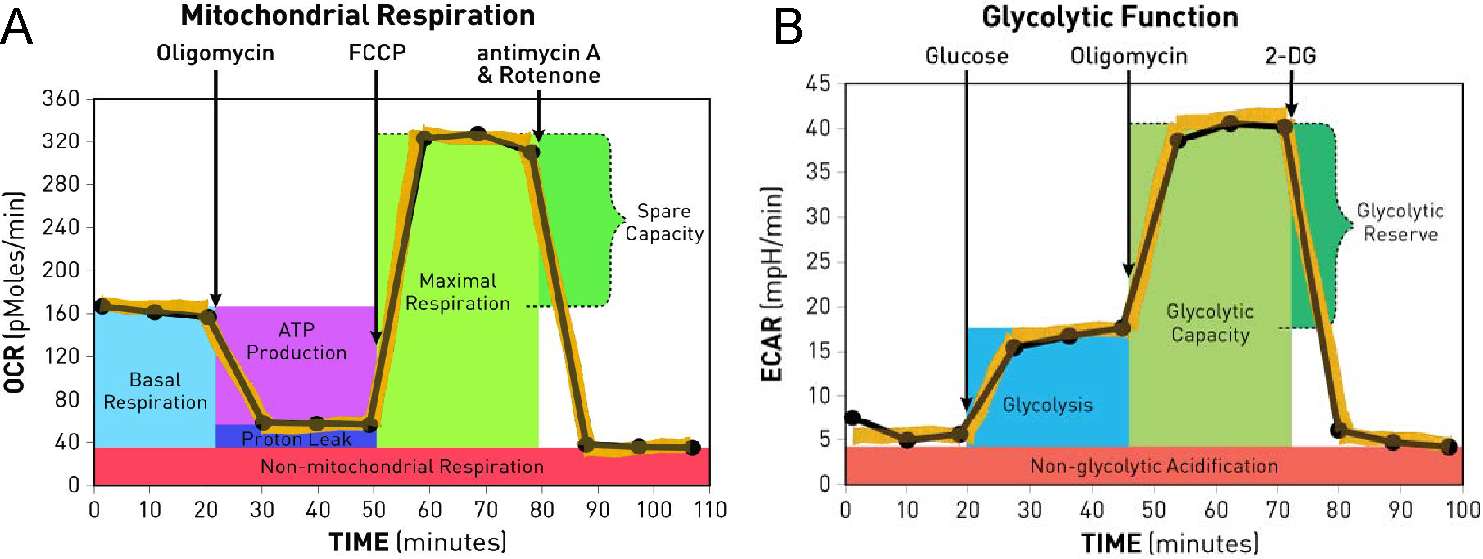

Supplement: Supplementary file 2 [file Image_1.TIF]

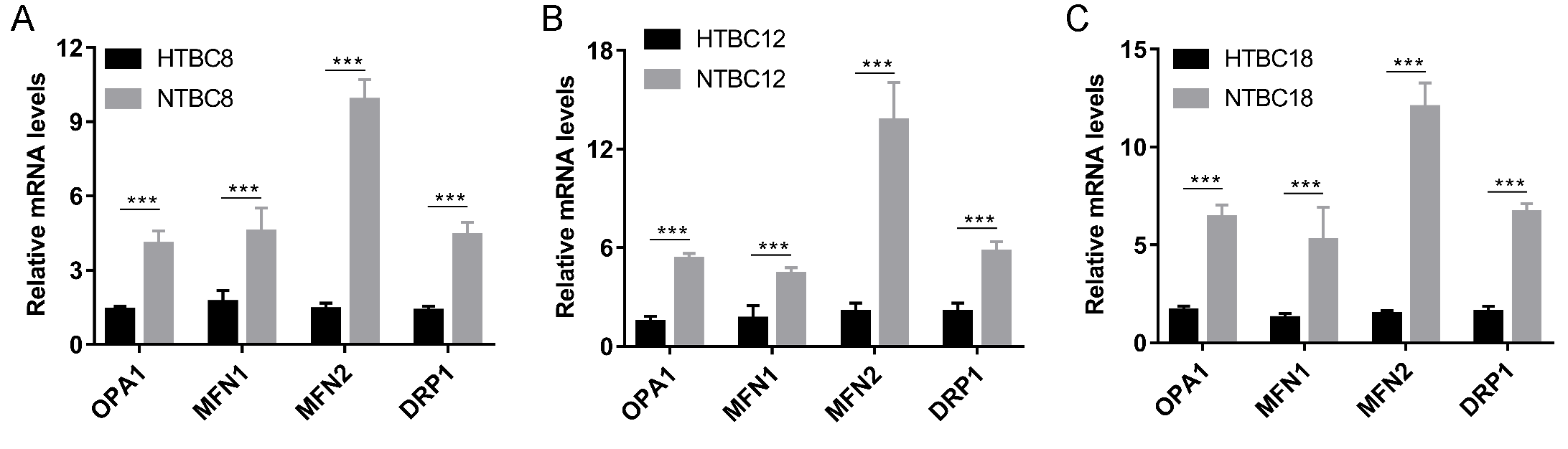

Supplement: Supplementary file 3 [file Image_2.TIF]

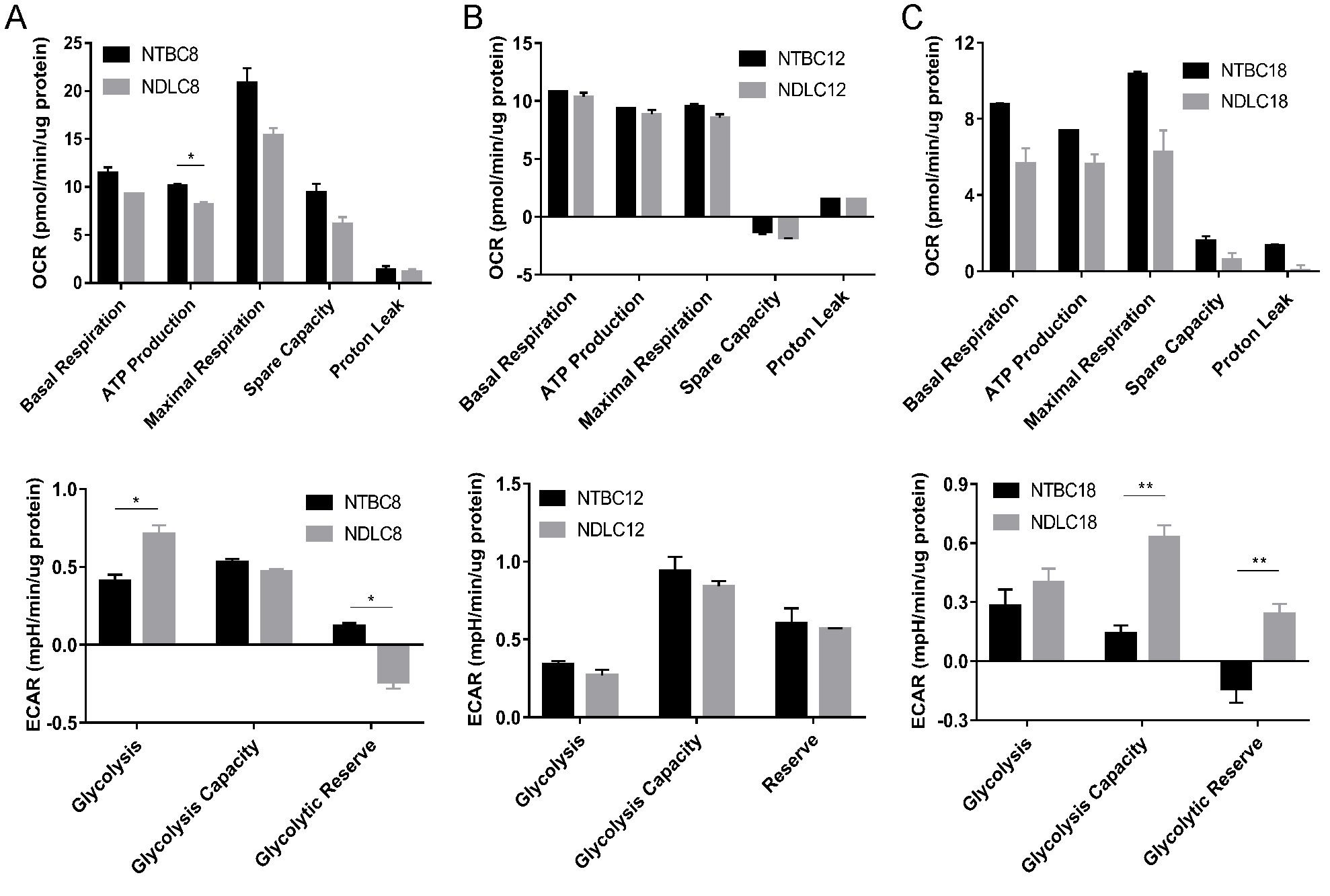

Supplement: Supplementary file 4 [file Image_3.TIF]

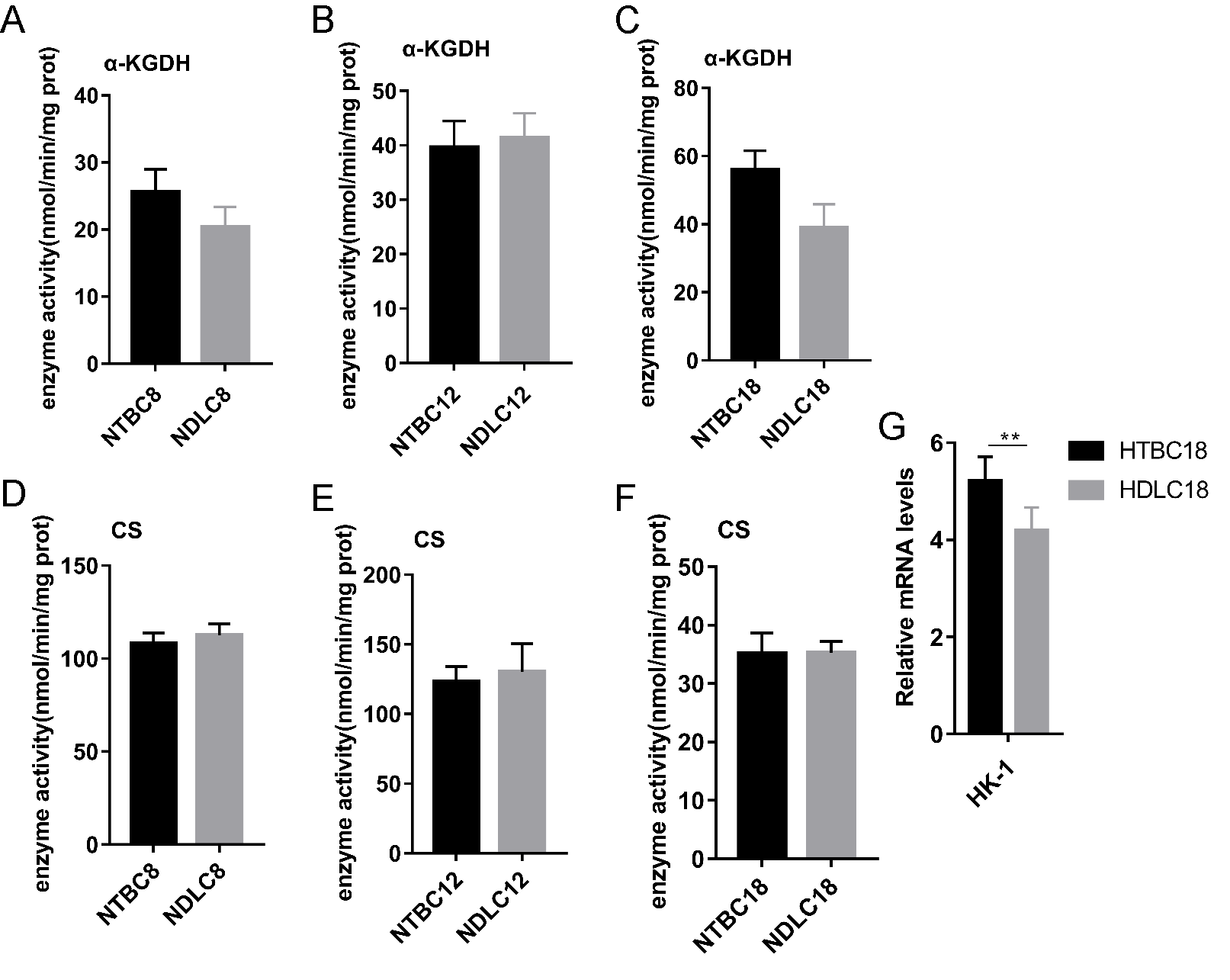

Supplement: Supplementary file 5 [file Image_4.TIF]

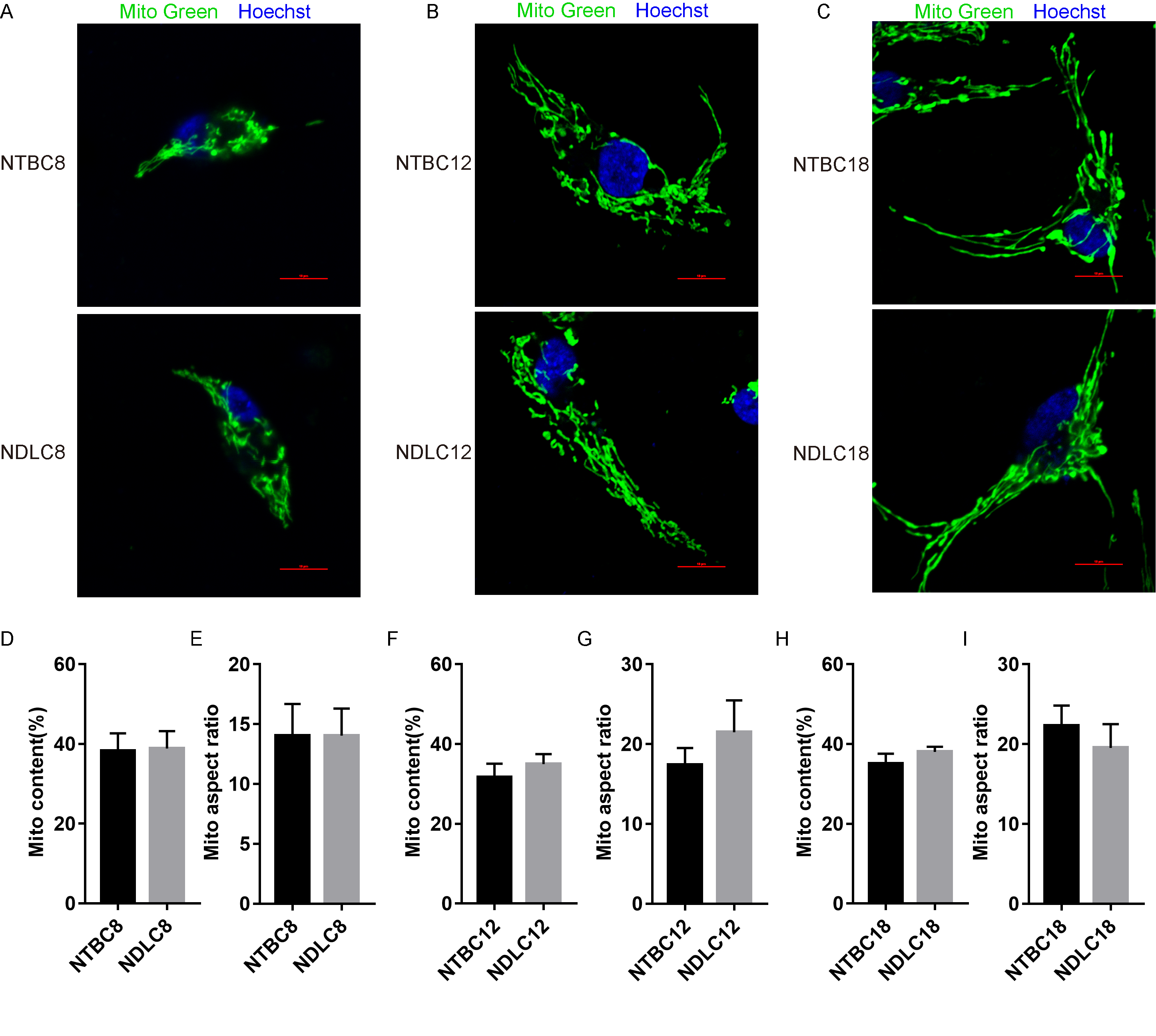

Supplement: Supplementary file 6 [file Image_5.TIF]

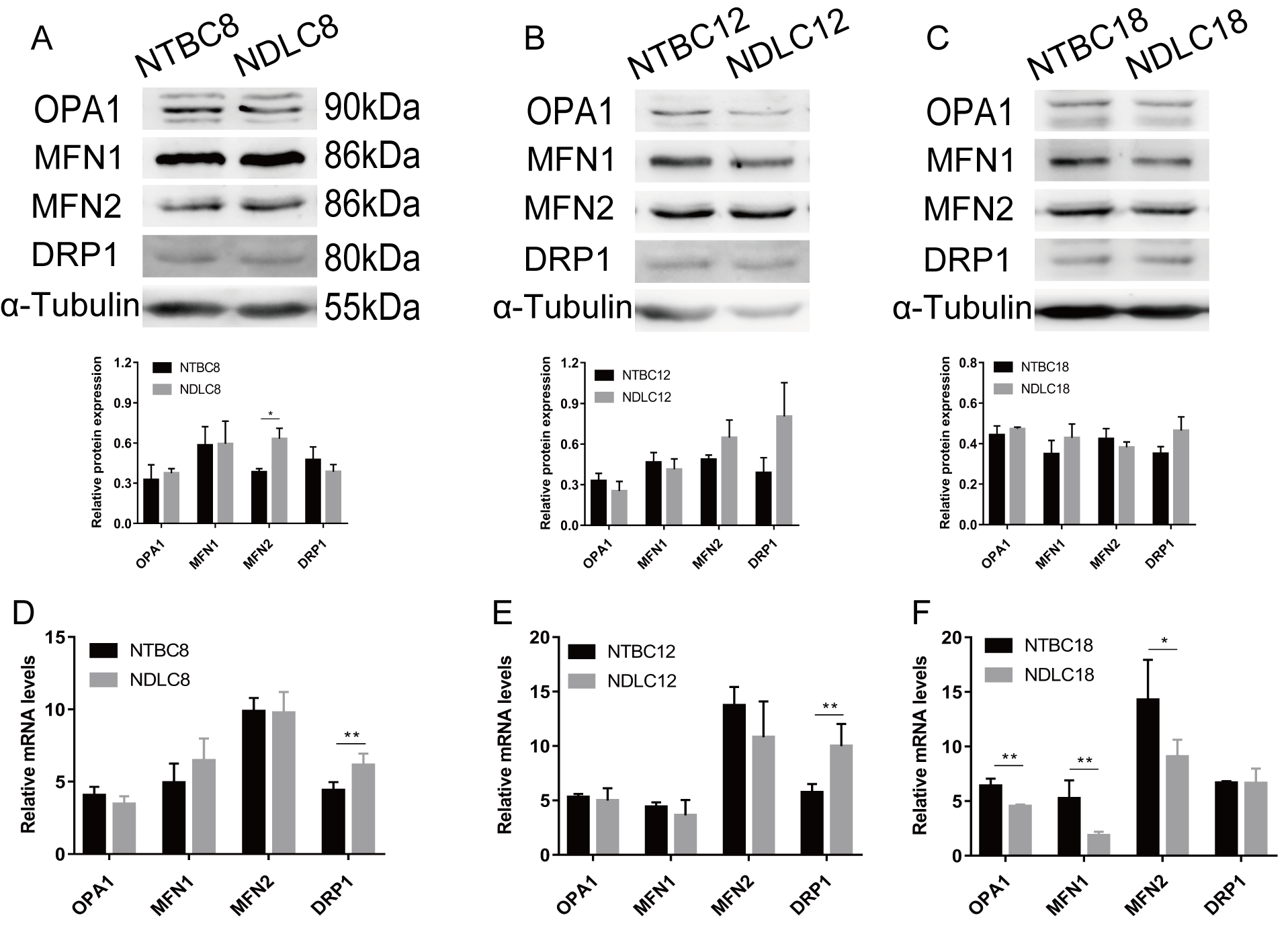

Supplement: Supplementary file 7 [file Image_6.TIF]

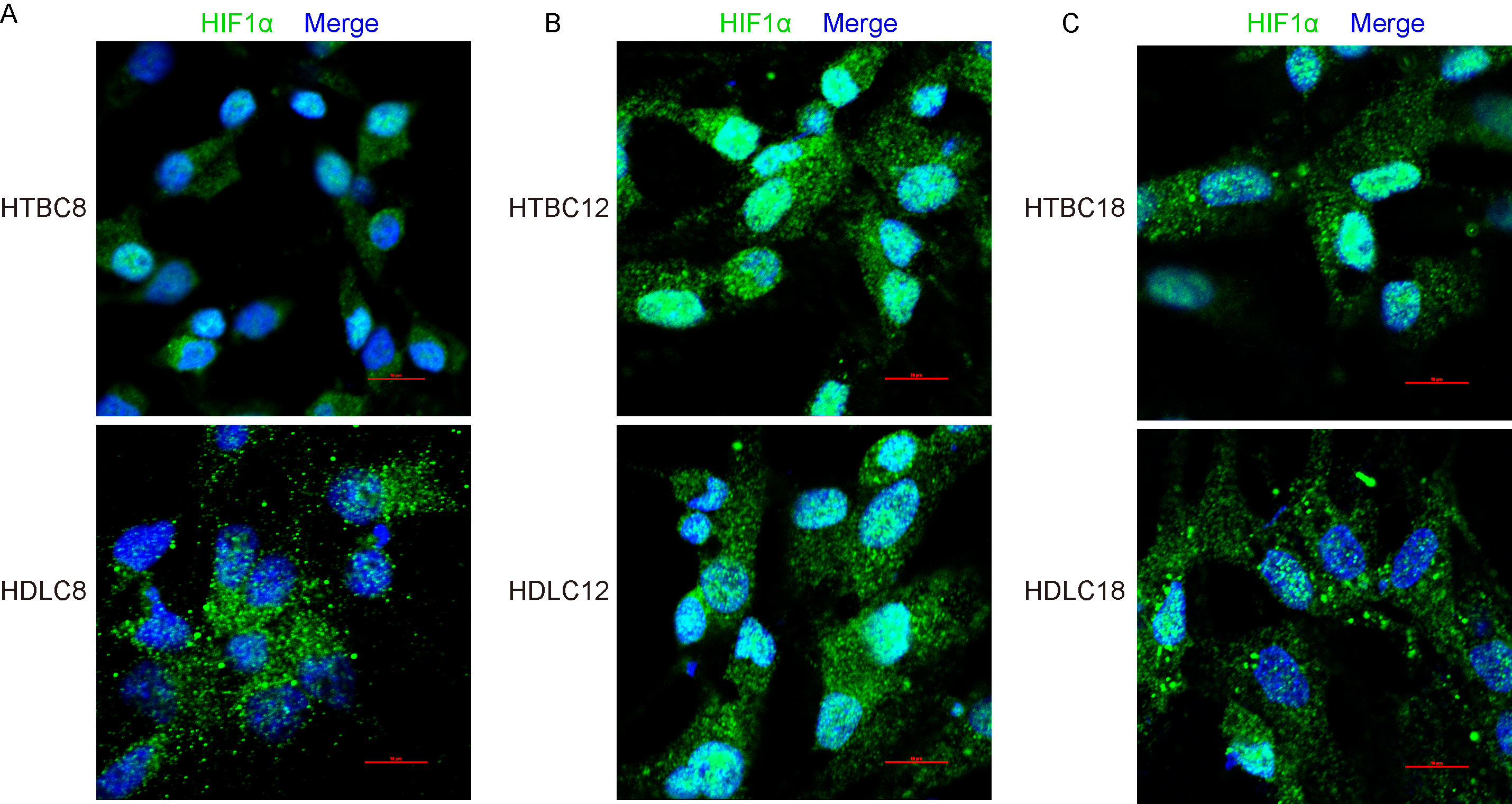

Supplement: Supplementary file 8 [file Image_7.TIF]
